# ATP/azobenzene-guanidinium self-assembly into fluorescent and multi-stimuli-responsive supramolecular aggregates

**DOI:** 10.1038/s42004-024-01226-y

**Published:** 2024-06-25

**Authors:** Olivier Abodja, Nadia Touati, Mathieu Morel, Sergii Rudiuk, Damien Baigl

**Affiliations:** 1 PASTEUR, Department of Chemistry, École Normale Supérieure, PSL University, Sorbonne Université, CNRS, 75005 Paris, France; 2 Chimie ParisTech, Université PSL, CNRS, Institut de Recherche de Chimie-Paris, PCMTH, 75005 Paris, France

**Keywords:** Self-assembly, Photochemistry, Self-assembly, DNA

## Abstract

Building stimuli-responsive supramolecular systems is a way for chemists to achieve spatio-temporal control over complex systems as well as a promising strategy for applications ranging from sensing to drug-delivery. For its large spectrum of biological and biomedical implications, adenosine 5’-triphosphate (ATP) is a particularly interesting target for such a purpose but photoresponsive ATP-based systems have mainly been relying on covalent modification of ATP. Here, we show that simply mixing ATP with AzoDiGua, an azobenzene-guanidium compound with photodependent nucleotide binding affinity, results in the spontaneous self-assembly of the two non-fluorescent compounds into photoreversible, micrometer-sized and fluorescent aggregates. Obtained in water at room temperature and physiological pH, these supramolecular structures are dynamic and respond to several chemical, physical and biological stimuli. The presence of azobenzene allows a fast and photoreversible control of their assembly. ATP chelating properties to metal dications enable ion-triggered disassembly and fluorescence control with valence-selectivity. Finally, the supramolecular aggregates are disassembled by alkaline phosphatase in a few minutes at room temperature, resulting in enzymatic control of fluorescence. These results highlight the interest of using a photoswitchable nucleotide binding partner as a self-assembly brick to build highly responsive supramolecular entities involving biological targets without the need to covalently modify them.

## Abstract (web summary)

Building stimuli-responsive supramolecular materials enables spatiotemporal control over complex systems, and is a promising strategy for a range of applications. Here, mixing adenosine 5’-triphosphate (ATP) with an azobenzene-guanidium compound possessing photodependent nucleotide binding affinity is shown to result in the spontaneous photo-reversible self-assembly of these compounds into micrometer-sized fluorescent aggregates that display dynamic responses to several chemical, physical and biological stimuli.

## Introduction

There is a general interest in developing stimuli-responsive systems due to their ability to modulate their functions in response to endogenous signals or external stimulations^1–3^. A widely used strategy to convert a passive system into a responsive one, consists in tethering a stimuli-responsive group, such as a photochrome, a pH-sensitive group or a redox species^4–8^ through adequate covalent modification. With the repertoire of known chemical reactions and coupling methods, this powerful approach can lead to virtually any user-defined system designed to respond to a desired stimulus. However, the necessity of covalently modifying the entity to be stimulated can alter its properties. It also requires to adjust the modification conditions for each system, making the approach case-specific. Stimulus-responsive self-assembly of molecular bricks through non-covalent interactions appears as an interesting alternative as it opens the possibility of dynamic actuation of inherently passive molecules without needing to modify them. This approach has already been demonstrated relevant for a large number of applications ranging from chemical sensors to drug-delivery systems^9^. The resulting stimuli-responsive self-assemblies are of particular interest when involving biological molecules to dynamically control their functions^10–13^. Among biologically active molecules, nucleotides and their analogs appear as particularly interesting targets for such a purpose^14^. For instance, adenosine 5’-triphosphate (ATP) is the universal energetic and ubiquitous molecule driving various biological processes and whose concentration can correlate with pathological conditions^15,16^. Moreover, ATP bears negatively charged phosphates as well as an aromatic π-conjugated adenine ring, allowing multiple electrostatic, π-π and hydrogen bonding interactions, thus making it prone to be engaged in supramolecular self-assembled systems^17,18^. Several stimuli-responsive ATP-based supramolecular systems have thus been developed but for only a few specific stimuli. One strategy consisted in exploiting ATP as an enzyme substrate to build enzyme-responsive assemblies. This was achieved in particular using phosphatase to control the fluorescence or chirality of the assemblies^19–22^. Another approach explored the chelating properties of ATP with metal ions to develop ion-responsive supramolecular structures but these investigations were limited to small-sized complexes^23–25^. Besides enzymes and metal ions, light constitutes a highly desired trigger to control self-assemblies due to its precise spatiotemporal control, tunability (wavelength, power) and remote character^26^. Light-responsive ATP-based systems to photochemically control the release of this nucleotide have been described but this involved photocleavable bonds, therefore precluding any photo-reversible response^27^. To our knowledge, photoreversible supramolecular assemblies involving unmodified ATP have yet to be demonstrated. To reach such a goal, azobenzene-containing molecules constitute promising co-assembly partners for their robustness and for a rapid and reversible *trans*-*cis* photoisomerization^28^. Cationic derivatives of azobenzene can be used in particular to electrostatically interact with negatively charged mono- or polynucleotides, a process that can be rendered photodependent through the polarity and/or geometric changes upon azobenzene *trans*-*cis* isomerization^29^. This was used for instance to photocontrol phenomena such as DNA compaction^30–38^, gene expression^34,39^, coacervation^40^, DNA intercalation^41,42^ or G-quadruplex formation^43^. With the objective to build extended photoreversible ATP assemblies in water, and thus limit the possibility of precipitation, we avoided classical azobenzene cationic surfactants^31,35,44–46^ and opted instead for a hydrophilic azobenzene partner, choosing guanidium as the cationic group for its strong affinity for nucleotide phosphate groups, including those of ATP^47–49^. We thus used AzoDiGua, a symmetric diguanidinium azobenzene derivative and photodependent DNA intercalator^41^, which was previously shown to co-assemble with guanosine monophosphate into photoswitchable and extended fluorescent crystals^50^. In this work we co-assembled AzoDiGua with ATP and observed the spontaneous formation in water of photo-reversible micrometer-sized supramolecular assemblies containing ATP. The self-assembled structures scattered light and exhibited fluorescence only in their assembled state, resulting in a reversible photoswitching of both turbidity and fluorescence. We also exploited the presence of ATP in the supramolecular structures to dynamically control their self-assembly by other stimuli, including metal ions and alkaline phosphatase (ALP) enzyme. With a combination of methods including optical microscopy, fluorescence spectroscopy, and electronic paramagnetic resonance (EPR), we characterized the reversibility, highly dynamic character and the switchable optical properties of these multi-stimuli-responsive ATP/azobenzene-guanidinium assemblies of a new kind.

## Results and discussion

### Preparation of ATP/AzoDiGua supramolecular aggregates

**Fig. 1:**
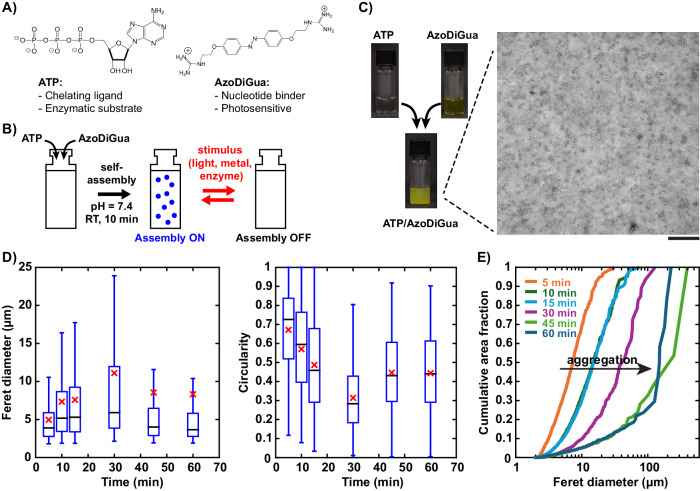
Spontaneous assembly of an azobenzene diguanidinium nucleotide binder (AzoDiGua) and adenosine 5’-triphosphate (ATP) into multi-stimuli-responsive micrometer-sized aggregates. **A** Molecular structure and essential characteristics of ATP (*left*) and AzoDiGua (*right*). **B** Scheme depicting the assembly set-up and resulting stimulus-responsive behavior. **C** Photographs of an ATP solution (*top left*), an AzoDiGua solution (*top right*) and the resulting mixture after 10 min of incubation at room temperature (*bottom*). Each tube has a diameter 1 cm and contains 500 µL of solution. The enlarged image is a transmission optical microscopy image of the aggregates (scale bar: 50 µm). **D**, **E** Morphological analysis of the supramolecular aggregates as a function of incubation time with a number of analyzed objects *n* = 839 (5 min), 1078 (10 min), 1122 (15 min), 644 (30 min), 507 (45 min), and 565 (60 min). **D** Box plots of Feret diameter (*left*) and circularity (*middle*). Blue box: range from first to third quartile; horizontal blue bars: minimum and maximum (outliers excluded); horizontal black line: median; red cross: mean. **E** Cumulative apparent area of the aggregates as a function of their Feret diameter, normalized by the total apparent area of the aggregates. The shift to the right (arrow) shows the progressive aggregation. All experiments are done using 1 mM ATP and 1 mM AzoDiGua at room temperature in 50 mM Tris HCl buffer solutions (pH = 7.4).

Figure 1 shows the main components and concept of this work. ATP (Fig. 1A, *left*) was used as a biologically active brick to be self-assembled. To ensure the formation of extended and light-responsive assembly, ATP was co-assembled with the photosensitive DNA intercalator AzoDiGua^41^, which contains two guanidinium groups with a strong affinity for phosphates and an azobenzene group enabling both π-π interaction and photoswitchability (Fig. 1A, *right*). We hypothesized that mixing ion- and enzyme-sensitive ATP with photosensitive AzoDiGua would lead to the spontaneous formation of multi-stimuli-responsive assemblies in water (Fig. 1B). The equimolar mixture of the two components ([ATP] = [AzoDiGua] = 1 mM) at physiological pH (7.4) and room temperature led to the immediate formation of a turbid solution (Fig. 1C, *left*). Optical microscopy observations revealed the presence of micrometer-sized entities that progressively aggregated with each other over time (Fig. 1C, *right*, Supplementary Fig. 1, Supplementary Movie 1). Image analysis on hundreds of individual aggregates showed that in the first 15 min, objects progressively increased in size with a Feret diameter (mean +/− sd on *n* analyzed objects) of 5.0 ± 3.2 µm (*n* = 839), 7.3 ± 6.3 µm (*n* = 1078) and 7.6 ± 6.8 µm (*n* = 1122) after 5, 10 and 15 min of assembly, respectively (Fig. 1D*left*). This was accompanied by a decreasing circularity (4π(area/perimeter^2^)) of 0.67 ± 0.2, 0.57 ± 0.22 and 0.46 ± 0.24, respectively, attributed to the aggregation of individual entities into more elongated structures (Fig. 1D*right*). At 30 min, the distribution became very broad with an average circularity 0.31 ± 0.18 (*n* = 644) denoting the presence of highly elongated aggregates. At longer assembly times, the suspension became highly heterogenous with the presence of a small number of aggregates of very large dimensions (Feret diameter >100 µm) leading to a mean Feret diameter (red crosses in Fig. 1D) highly shifted from most of the values, and even above the third quartile of the distribution. Interestingly, many small aggregates coexist with these large structures as shown by the median values (horizontal black lines in Fig. 1D) at 45 min (4.0 µm, *n* = 507) and 60 min (3.7 µm, *n* = 565) that are comparable to that for shorter assembly times (3.9, 5.1 and 5.3 µm for 5, 10 and 15 min, respectively). To better quantify whether ATP/AzoDiGua was mainly assembled in the form of dispersed micrometric entities or in contrast involved in a few very large aggregates, we measured the apparent area (projected in the xy plane) occupied by all objects having a Feret diameter smaller than a given value and normalized it by the apparent area of all detected objects. Figure 1E displays this fraction as a function Feret diameter for different assembly times. This analysis clearly shows that, despite the remaining presence of many small entities all along the assembly process with a rather constant median Feret diameter around 4–5 µm, most of the self-assembled material progressively evolved from well-defined µm-sized entities at low assembly times (less than 15 min) to very large aggregated structures (>100 µm) sedimenting in solution after 30 min of assembly. Assemblies of similar size and morphology were recently reported for ATP mixed with oppositely charged ammonium- or guanidinium-rich polycations, highlighting the interest of exploiting electrostatic interactions, though the resulting structures were not responsive^51^. To evaluate the role of the phosphate groups in the ATP-AzoDiGua self-assembly process, we performed the same experiments using ATP analogs, and especially the monophosphate (adenosine monophosphate, AMP) and the diphosphate (adenosine diphosphate, ADP) derivatives (Supplementary Fig. 2). After 10 min of self-assembly with AzoDiGua (1 mM), a markedly different behavior was observed between AMP, ADP and ATP. With AMP (1 mM), the solution remained transparent and no aggregate could be detected by optical microscopy, in agreement with results previously obtained after a thermal annealing treatment^50^. In contrast, compared to AMP, ADP (1 mM) led to the formation of supramolecular aggregates of similar morphology (circularity = 0.64 ± 0.2, *n* = 1044) but with a Feret diameter distribution shifted to lower values with smaller mean (4.3 ± 2 µm) and median (3.7 µm) values (Supplementary Fig. 3), leading to a less turbid solution than that with ATP (Supplementary Fig. 2*insets*). This evolution not only emphasizes the crucial role of the phosphate groups for the supramolecular self-assembly to occur but provides also a way to tune the aggregate characteristics by the number of phosphate per AzoDiGua partner. When using ATP at an acidic pH (2.0 instead of 7.4), the ATP/AzoDiGua mixture solution remained transparent and no extended supramolecular structures could be detected by optical microscopy (Supplementary Fig. 4), which was attributed to a lower ionization degree of ATP, its possible hydrolysis at acidic pH or a combination of both effects^17^. All these results show the important role of the electrostatic interactions between the negatively charged phosphate groups of ATP and the cationic guanidinium moieties of AzoDiGua. To have further insights into the molecular interactions between AzoDiGua and ATP at a low concentration where microscopic aggregation did not occur, a spectroscopic titration of AzoDiGua by ATP was performed. We observed a hypochromic effect on the π-π * transition of AzoDiGua at 360 nm with increasing amounts of ATP (Supplementary Fig. 5). This effect is in agreement with the establishment of π-π interactions between ATP and AzoDiGua, which are thus expected to be involved in the formation of the supramolecular aggregates at higher concentrations. In summary, ATP and AzoDiGua spontaneously self-assembled at room temperature and physiological pH into supramolecular aggregates through combined effects of electrostatic interactions and π-π stacking. These aggregates reached a size of a few micrometers in only a few minutes, before slowly evolving into larger sedimenting aggregates after 30 min of assembly. To characterize their properties, including their reversibility and dynamic character, we fixed their composition ([ATP] = [AzoDiGua] = 1 mM) and assembly time to 10 min before studying their photoluminescent properties and response to the application of different physical, chemical and biological stimuli.

### Fluorescence of the supramolecular assembly

**Fig. 2:**
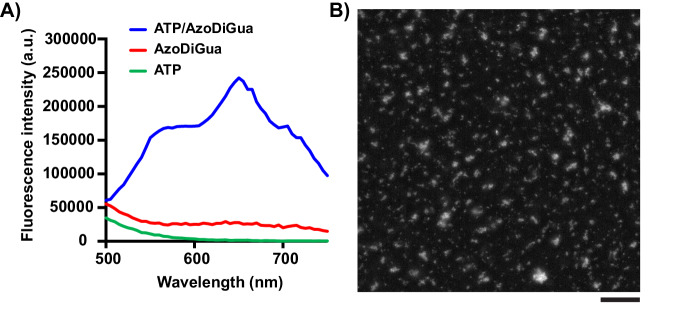
ATP/AzoDiGua aggregates are fluorescent upon assembly of non-fluorescent constituents. **A** Fluorescence emission spectra (Exc: 400 nm) of ATP alone (1 mM), AzoDiGua alone (1 mM), and the resulting mixture after 10 min of assembly. **B** Fluorescence microscopy image (Exc: 475/20 nm; Em: 500–800 nm) of the supramolecular ATP/AzoDiGua aggregates shown in Fig. 1C*right*. The scale bar is 50 µm. All experiments are done at room temperature in 50 mM Tris HCl buffer solutions (pH = 7.4).

The presence of the AzoDiGua in the aggregates led us to explore whether photoluminescent properties could be acquired by these assemblies through the aggregation-induced fluorescence mechanism of azobenzene^52–57^. Using fluorescence spectroscopy, we found that ATP/AzoDiGua aggregates were broadly absorbing in the range 320–460 nm, displaying a reproducible photoluminescent emission regardless of the excitation wavelength (Supplementary Fig. 6) with a maximum at around 650 nm (Fig. 2A). Notably, neither ATP nor AzoDiGua alone was significantly absorbing for a photoluminescent emission at 650 nm (Supplementary Fig. 7) nor photoluminescent when excited at 400 nm (Fig. 2A, Supplementary Fig. 8). Moreover, the same system prepared with ADP or AMP, instead of ATP, displayed weak photoluminescence for ADP/AzoDiGua where small aggregates formed, and no detectable signal for AMP/AzoDiGua where no assembly was observed (Supplementary Fig. 9). All these results show that the self-assembly between ATP and AzoDiGua was instrumental in the emergence of the photoluminescence from the ATP/AzoDiGua suspension. It was shown in the past that restricting the conformation of AzoDiGua in a co-assembled crystal hindered its rotation/inversion relaxation upon photoexcitation leading to a neat enhancement of fluorescence^50^, with excitation/emission properties similar to that obtained in the ATP/AzoDiGua system. We thus ascribed the photoluminescence of the ATP/AzoDiGua assemblies to a similar aggregation-induced fluorescence mechanism leading to enhanced fluorescence of AzoDiGua when engaged in the supramolecular aggregates. Fluorescence microscopy (Fig. 2B) confirmed that the fluorescence emission was mainly localized at the position of the micrometer-sized ATP/AzoDiGua aggregates. Therefore, ATP and AzoDiGua self-assembled to form fluorescent aggregates while their individual components, AzoDiGua and ATP alone, were not intrinsically fluorescent in solution. We then studied how different stimuli could dynamically control the assembly and therefore affect its aggregation-induced fluorescence properties.

### Photoresponse of ATP/AzoDiGua assembly

**Fig. 3:**
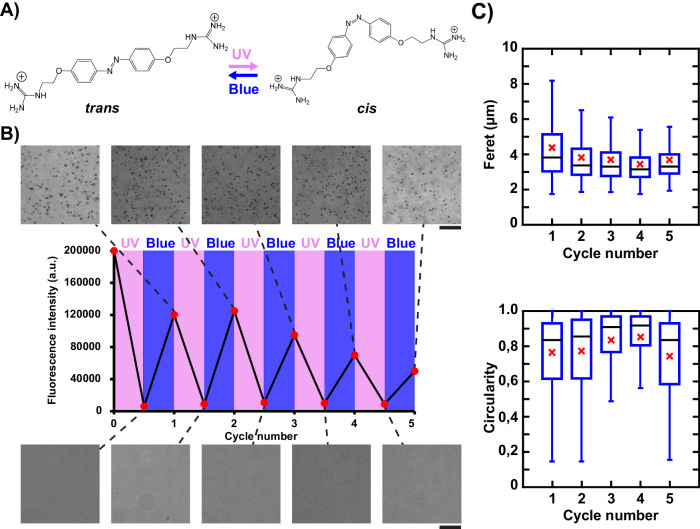
Photoreversible assembly of fluorescent ATP/AzoDiGua aggregates. **A** Photoisomerization scheme of AzoDiGua upon UV and blue irradiation. **B** Transmission optical microscopy images and fluorescence intensity (Exc: 400 nm; Em: 650 nm) of the ATP/AzoDiGua suspension after consecutive cycles of UV (395/25 nm, 26 mW·cm^−2^) for 3 min and blue irradiation (480/30 nm, 40 mW·cm^−2^) for 3 min. The initial suspension results from the self-assembly of ATP (1 mM) and AzoDiGua (1 mM) for 10 min. The scale bars are 20 µm. **C** Box plots of Feret diameter (top) and Circularity (bottom) of the supramolecular aggregates obtained after each UV/blue cycle displayed in **B**. The number of analyzed objects is *n* = 659, 1012, 1044, 994 and 817 after 1, 2, 3, 4 and 5 cycles, respectively. Blue box: range from first to third quartile, horizontal blue bars: minimum and maximum (outliers excluded); horizontal black line: median; red cross: mean. All experiments are done at room temperature in 50 mM Tris HCl buffer solutions (pH = 7.4).

Since the aggregates resulted from the co-assembly of AzoDiGua with ATP, we next investigated the photoreversibility of ATP-based supramolecular assemblies. Indeed, pure AzoDiGua is able to reversibly photoisomerize between *trans* and *cis* conformations upon UV and blue light irradiation (Fig. 3A)^41^. To characterize the photoresponse of the supramolecular aggregates, we first prepared the ATP/AzoDiGua aggregates in the regular conditions ([ATP] = 1 mM, [AzoDiGua] = 1 mM, 10 min incubation at room temperature) using pure *trans*-AzoDiGua as a starting material and exposed them to successive irradiations by UV (395/25 nm, 26 mW·cm^−2^) for 3 min and blue (480/30 nm, 40 mW·cm^−2^) for 3 min. The first UV application resulted in the progressive disassembly of the supramolecular aggregates until their complete disappearance in less than 1 min (Supplementary Movie 2, Supplementary Fig. 10*middle*). This disassembly is reminiscent of the photoresponse observed when AzoDiGua interacted with guanosine monophosphate (GMP) to form photoswitchable crystals^50^. In that case, we showed that the UV-induced isomerization of AzoDiGua into the *cis* form resulted in the disruption of the geometry-dependent supramolecular interactions (π-π and hydrogen bond interaction network) leading to the disassembly of the crystals under UV light. Additionally, the *trans*- to *cis*- isomerization is accompanied by an increase of AzoDiGua polarity. In the case of amphiphilic azobenzene molecules, this effect has been demonstrated to decrease the efficacy of cooperative binding with oppositely charged partners such as DNA^35^. In the present case, both the geometry and polarity change when AzoDiGua switches from the *trans* to the *cis* isomer can disfavor the π-π stacking and electrostatic interactions at the origin of the supramolecular aggregates formation, resulting in efficient UV-induced disassembly. The subsequent blue irradiation led to an enrichment in *trans*-AzoDiGua isomer and the recovery of the assembly in less than 1 min (Supplementary Movie 2, Supplementary Fig. 10*right*). This was accompanied by a photocontrol of the optical properties of the ATP/AzoDiGua suspension. The first UV irradiation led to the loss of the suspension turbidity due to the vanishing of the light-scattering entities (Supplementary Fig. 10*middle*) and appearance of the characteristic color of the *cis*-AzoDiGua isomer enriching the solution (Supplementary Fig. 11*right*). Similarly, the subsequent exposure to blue irradiation induced a change of the optical aspect of the suspension which became turbid again, due to the reappearance of the micrometer-sized ATP/AzoDiGua aggregates (Supplementary Fig. 10*right*). Note that, compared to the initial suspension composed of pure *trans*-AzoDiGua, the suspension after UV and blue irradiation was less turbid and the aggregates were smaller (mean Feret diameter: 4.4 ± 2.2 µm, *n* = 659) and less numerous, which is explained by the incomplete *trans*-isomer enrichment upon blue irradiation in the photostationary state^41^.

This successive disassembly/reassembly of the ATP/AzoDiGua aggregates upon UV/blue irradiation was then successfully repeated for five complete consecutive cycles of UV (395/25 nm, 26 mW·cm^−2^) for 3 min and blue (480/30 nm, 40 mW·cm^−2^) for 3 min (Fig. 3B). Complete disappearance was observed after each UV irradiation (Fig. 3B*bottom*) while aggregates reproducibly reassembled after blue irradiation (Fig. 3B top) with similar size and shape distribution regardless of the number of applied cycles (Fig. 3C). This was accompanied by a dynamic and reversible photocontrol of the suspension fluorescence correlating with the assembly state, i.e., high intensity for the blue-induced assembly and low intensity after UV-induced disassembly, respectively (Fig. 3B*middle*). Again, the fluorescence after the first UV/blue cycle was lower than in the initial solution (Supplementary Fig. 12) due to a lower fraction of *trans*-AzoDiGua in the photostationary state compared to the pure *trans* solution before irradiation. After the first cycle, a smaller but progressive decrease of fluorescence intensity was also observed with an increase in the number of applied UV/blue cycles indicating either partial photobleaching upon successive irradiation or progressive loss of emitting materials, due to the sedimentation of the largest aggregates. Since the morphological characteristics of the aggregates were not significantly affected along the different photo-stimulations (Fig. 3C), photobleaching might thus be the predominant mechanism. By exploiting the co-assembly between ATP and AzoDiGua, we achieved both photoswitchable and aggregation-induced fluorescence of ATP/AzoDiGua aggregates leading, to our knowledge, to the first photoswitchable micro-meter-sized and fluorescent ATP-based assembly.

### ATP/AzoDiGua assembly response to metal ions

**Fig. 4:**
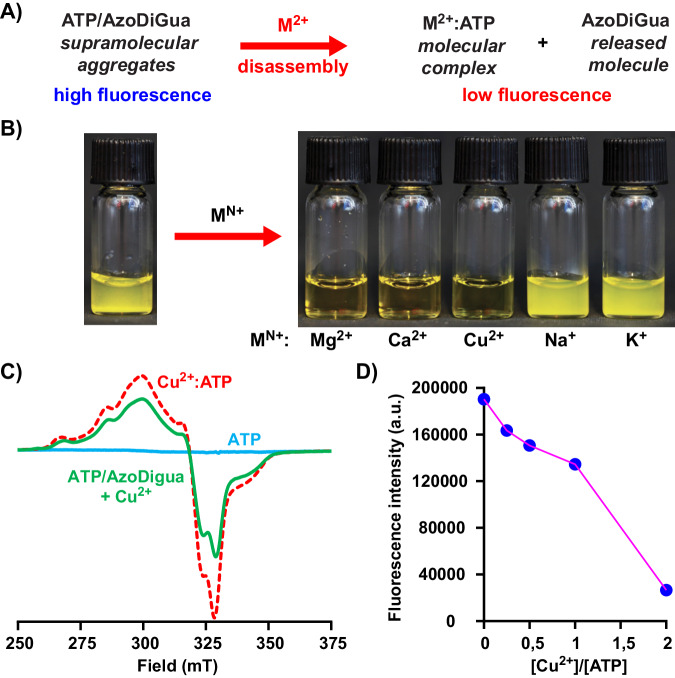
ATP/AzoDiGua aggregates are cation-responsive and valence-selective. **A** Scheme of metal ion-triggered ATP/AzoDiGua disassembly by ATP chelation with di- and monovalent cations. **B** Photographs of ATP/AzoDiGua suspensions before (left) and after (right) addition of different metal cations (M^N+^) with a ratio [M^N+^]/[ATP] = 2. **C** Electron Paramagnetic Resonance (EPR) spectra at *T* = 110 K of ATP alone (1 mM) (blue solid line), mixture of Cu(NO_3_)_2_ (2 mM) and ATP (1 mM) (dashed line), and ATP/AzoDiGua aggregates after disassembly by adding Cu(NO_3_)_2_ at a ratio [Cu^2+^]/[ATP] = 2 (green solid line). **D** Fluorescence intensity (Exc: 400 nm; Em: 650 nm) of ATP/AzoDiGua suspension after addition of Cu(NO_3_)_2_ as a function of the ratio [Cu^2+^]/[ATP]. The solid line between symbols (data points) is guide for the eye. For all experiments, the initial suspension results from the self-assembly of ATP (1 mM) and AzoDiGua (1 mM) for 10 min. All experiments are done at room temperature in 50 mM Tris HCl buffer solutions (pH = 7.4).

To expand the stimulus-responsivity of the photoswitchable aggregates, we exploited the chelating properties of ATP to study the possibility to control this assembly with ions. Knowing the strong affinity between ATP and divalent metal ions^23,58^, our hypothesis was that the addition of such cations could favor the ATP-metal complex formation, leading to the disassembly of the aggregates and the decrease of their fluorescence (Fig. 4A). Hence, we started from the preformed ATP/AzoDiGua aggregates and added different metal cations (M^N+^) to ensure a final molar ratio [M^N+^]/[ATP] = 2. Interestingly, it was found that the turbidity of the solution immediately vanished for all tested divalent cations (Mg^2+^, Ca^2+^, and Cu^2+^) but remained for the monovalent ones (Na^+^, K^+^) (Fig. 4B), showing that the ion-triggered disassembly was valence-selective. The color due to the presence of *trans*-AzoDiGua (Supplementary Fig. 11*left*) remained upon ion-triggered disassembly, confirming that the response was mediated by ATP chelation and not by a change of AzoDiGua isomerization state. The mechanism of the metal-mediated disassembly of the aggregates was further investigated in the case of Cu^2+^ using EPR spectroscopy, keeping all concentrations identical to that used for the assembly/disassembly experiments ([ATP] = 1 mM ; [AzoDiGua] = 1 mM ; [Cu^2+^] = 2 mM). The starting solution of copper (Cu(NO_3_)_2_, 2 mM) showed a typical EPR signal of Cu^2+^ (S = 1/2) centered at a value of *g*-factor near 2.08 (Supplementary Fig. 13). After addition of ATP solution (2 mM), this signal shifted to lower fields and displayed a new marked shouldering, suggesting interaction between Cu^2+^ and ATP upon chelation^59,60^. Contrary to Cu^2+^/ATP solution mixture, the EPR signal of Cu^2+^ did not evolve upon addition of AzoDiGua (1 mM, Supplementary Fig. 13B). EPR measurements were carried out also on ATP (1 mM) and AzoDigua (1 mM) solutions and no signal was observed (Fig. 4C, Supplementary Fig. 13). Like ATP alone and AzoDiGua alone, AzoDiGua/ATP aggregates did not have any EPR signal. However, their disassembly upon addition of Cu^2+^ was accompanied by the appearance of a paramagnetic signal displaying the same features as the one of Cu^2+^/ATP mixture, with the characteristic peaks at nearly the same positions (Fig. 4C) and the same *g*-factor (≈2.08). This shows that the disassembly of ATP/AzoDiGua aggregates upon Cu^2+^ addition was due to the competitive complexation between ATP and Cu^2+^. Enlarging the spectra in the region 240–280 mT revealed the emergence of a small additional peak and a slight shift of the peaks between pure Cu^2+^/ATP mixture and ATP/AzoDiGua aggregates disassembled by Cu^2+^ (Supplementary Fig. 14). ATP and AzoDiGua alone being EPR silent, we attribute this effect to the appearance of an additional species in which Cu^2+^ is involved and probably associated to both ATP and AzoDiGua. This species is a minority one and only marginally affects the spectrum of the Cu^2+^/ATP complexes formed upon disassembly of the ATP/AzoDiGua aggregates. Optical microscopy observations of the ATP/AzoDiGua aggregates ([ATP] = 1 mM ; [AzoDiGua] = 1 mM) exposed to increasing amounts of Cu^2+^ evidenced a gradual disassembly with an increase in [Cu^2+^] and showed that an excess of Cu^2+^ (2 mM) was necessary to ensure full and permanent disassembly (Supplementary Fig. 15). This ion-stimulated disassembly was then used to control the fluorescence of ATP/AzoDiGua co-assembly (Fig. 4D, Supplementary Fig. 16), showing either a way to chemically adjust the fluorescence of the system or an original method to titrate the presence of divalent cations (here, Cu^2+^) by fluorescence. After disassembly for a ratio [Cu^2+^]/[ATP] = 2, further addition of ATP (3 mM) led to the re-assembly of ATP/AzoDiGua aggregates and appearance of suspension turbidity (Supplementary Fig. 17), emphasizing the reversibility of the supramolecular aggregates and the role of free ATP for the assembly to occur. By exploiting the chelating properties of ATP and its strong and specific affinity for divalent cations, we demonstrated that ATP-AzoDiGua aggregates were cation-responsive and valence-selective, offering the possibility not only to dynamically control both their assembly/disassembly behavior and their fluorescence through a chemical stimulus but also to envision the principle of a divalent-specific ionic sensor.

### Enzymatic response of ATP/AzoDiGua assembly

**Fig. 5:**
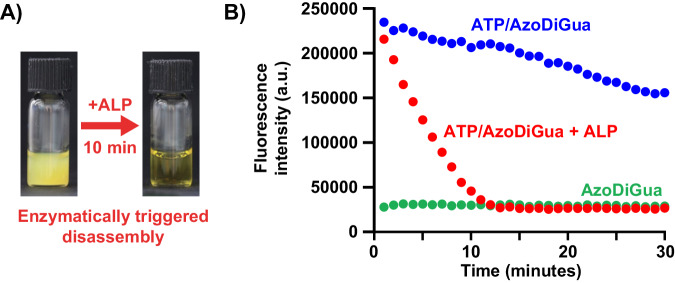
Enzymatic control of the ATP/AzoDiGua assembly and fluorescence. **A** Photographs of ATP/AzoDiGua suspension before (left*)* and after addition of alkaline phosphatase (ALP, 0.025 µg/µL) for 10 min (right). **B** Fluorescence intensity (Exc: 400 nm; Em: 650 nm) as a function of time for the ATP/AzoDiGua suspension without (blue symbols) or with (red symbols) added ALP to ATP/AzoDiGua suspension at *t* = 0, or for AzoDiGua alone (1 mM, green symbols) with added ALP at *t* = 0. For all experiments, the initial ATP/AzoDiGua suspension results from the self-assembly of ATP (1 mM) and AzoDiGua (1 mM) for 10 min. All experiments are done at room temperature in 50 mM Tris HCl buffer solutions (pH = 7.4).

We finally explored the use of the catalytic activity of an enzyme as a stimulus to trigger the disassembly of the ATP/AzoDiGua aggregates. Alkaline phosphatase (ALP) was chosen as an enzymatic cleavage agent for its ability to catalytically hydrolyze ATP into adenosine and three equivalents of monophosphate groups in mild conditions. We exploited this property and used ALP as a trigger to dynamically control ATP/AzoDiGua self-assembly. Adding ALP to the preformed ATP/AzoDiGua aggregates ([ATP] = 1 mM, [AzoDiGua] = 1 mM) led to their complete disassembly in 10 min (Supplementary Fig. 18). This disassembly was accompanied by a loss of turbidity of the ATP/AzoDiGua solution after adding the enzyme to the mixture (Fig. 5A). When the same experiment was performed with a previously denatured ALP (treatment at 90 °C), the suspension turbidity remained and no disassembly was observed (Supplementary Fig. 19), confirming that ALP-triggered disassembly was due to ATP hydrolysis by the active enzyme. The enzymatic disassembly of the ATP/AzoDiGua aggregates was then followed by fluorescence (Fig. 5B). Without enzyme, the fluorescence of the ATP/AzoDiGua aggregates gradually decreased over time during the first 30 min of measurement, i.e., before the formation of very large (>100 µm) aggregates (Fig. 1D). This decrease was thus primarily attributed to photobleaching. In contrast, the addition of ALP induced a much steeper decrease of fluorescence down to the level corresponding to that of free AzoDiGua after about 10 min of reaction (Fig. 5B), in agreement with the complete disassembly of the aggregates by the enzyme and concomitant release of free AzoDiGua. These results thus demonstrate how both aggregation-induced fluorescence of azobenzene and the characteristics of a self-assembled structure can be dynamically controlled through the action of an active enzyme having as a substrate one of two co-assembled partners.

## Conclusions

Adenosine 5’-triphosphate (ATP) is involved in many biological and biotechnological processes, making its dynamic actuation, especially by light, a valuable challenge for a broad range of applications of fundamental and/or practical interest. Instead of the conventional approach involving a covalent modification to render it stimulus-responsive, we have described an original strategy where ATP is associated with a photosensitive nucleotide binder called AzoDiGua to form multi-stimuli-responsive supramolecular structures through non-covalent interactions. AzoDiGua is a water-soluble azobenzene molecule with two guanidinium groups at its extremities and known as a photosensitive DNA intercalator. Here, we exploited its phototunable ability to interact with nucleotides through π-π and electrostatic interaction to build a new kind of photoresponsive co-assembly using ATP as a co-assembly partner. We showed in particular that simply mixing AzoDiGua and ATP led to the spontaneous assembly of the two partners into micrometer-sized aggregates in a few minutes in water. This assembly was found to be robust and also occurred in complex media containing a large number of biomolecules, including DNA and proteins, as well as in conditions compatible with cell culture (Supplementary Note 1, Supplementary Fig. 20). The benefits of such an approach are manifold. First, the approach allows to dynamically control the assembly/release of ATP by light without having to modify it, and in mild conditions (physiological pH, room temperature, ATP concentrations similar to intracellular one), making the approach particularly robust, prone to generalization to other targets and opening interesting perspectives for in situ non-invasive actuation. Second, the non-covalent assembly allows to maintain the stimulus-responsiveness of the individual bricks in the supramolecular structure. For instance, AzoDiGua remained photoswitchable and enabled photoreversible assembly/disassembly of the aggregates by short UV/blue irradiation pulses for several consecutive cycles. Similarly, intrinsic chelating properties and enzymatic reactivity of ATP have been exploited to dynamically trigger fast disassembly of the structures by the action of metal cations or enzymes. Third, fluorescence appeared as an emerging property arising from the self-assembly itself. Indeed, although ATP and AzoDiGua have only very weak fluorescent properties when they are individually dispersed in solution, their co-assembly led to significantly fluorescent superstructures. Such aggregation-induced fluorescence of azobenzene is a well-known phenomenon but is in many cases obtained with pure azobenzene compounds loosing most of their photoswitchability in the aggregated state. Here, the co-assembly with ATP allowed us not only to combine both aggregation-induced fluorescence and photoswitchability in the same system, but also to realize multi-stimuli-responsive fluorescence. For instance, the fluorescence of the ATP/AzoDiGua supramolecular aggregates was successfully and dynamically controlled by various physical (light), chemical (metal cations) and biological (enzyme) stimuli. This not only further evidenced the dynamic control over the self-assembly achieved by these different stimuli but also provides opportunities for the development of sensing strategies for detecting ATP, analyzing the presence of metal cations with valence-selectivity, or assessing enzymatic activity. Demonstrated here with two particular bricks, ATP and AzoDiGua, we think this co-assembly strategy could be explored with other nucleotide or DNA/RNA targets and/or other stimuli-responsive DNA intercalators to build highly dynamic and multifunctional supramolecular structures in a particularly robust, user-friendly and multi-purpose way.

## Methods

### Preparation of the ATP/AzoDiGua self-assembly system at physiological pH

A reaction solution was prepared at room temperature by adding in this order Tris HCl buffer, water, and proper amounts of a freshly prepared solution of AzoDiGua (5 mM in water) and of a mother solution of ATP (50 mM in water) and mixed by pipetting up and down several times. For most experiments, the final concentrations were [ATP] = 1 mM and [AzoDiGua] = 1 mM in 50 mM Tris-HCl (pH = 7.4). The resulting mixture was left at room temperature for an incubation time of about 10 min unless otherwise specified.

### Preparation of other solutions

For ADP/AzoDiGua and AMP/AzoDiGua self-assembly studies, we used the same procedure as for ATP/AzoDiGua except for the ATP solution that was replaced with the corresponding ADP or AMP solution. All solutions were prepared in Tris-HCl buffer at a final concentration of 50 mM (pH = 7.4) except for the study at acidic pH (Supplementary Fig. 4). In that case, a calibration buffer (pH = 2.00, Mettler Toledo) was mixed with water and stock aqueous solutions of ATP and AzoDiGua to final concentrations of [ATP] = [AzoDiGua] = 1 mM. The volume of the calibration buffer represented half of that of the final solution. The pH of the final solution was measured (SevenCompact, Toledo pH meter) to remain at pH = 2.

### Microscopy observations

100 μL of the reaction medium was put into a well of a 96-well plate (Microplates Greiner bio one F-bottom model) and placed on the stage of an inverted microscope (Axio Observer Z1, Zeiss) equipped with a sCMOS camera (Zyla 4.2plus, Teledyne Photometrics) and a Xenon light source (HXP 120, Zeiss). The microscope was used either in a transmission mode, or in reflection mode for fluorescence imaging using a specific optical filter set with a large emission wavelength range (Excitation: 475/40 nm; Dichroic: 500 nm ; Emission: longpass 515 nm).

### Microscopy image analysis

Images from optical transmission microscopy were analyzed using ImageJ. Briefly, images were segmented using a fixed intensity threshold to detect the darker regions. Binary images were then cleaned with a Despeckle filter and by binary closing. Area, Feret diameter and circularity were measured for each separate region of at least 1 µm^2^.

### Fluorescence spectroscopy

All measurements were carried out on a Tecan Spark fluorimeter plate-reader. Excitation (emission: 650 nm) and emission (excitation: 400 nm) spectra were recorded using 100 µL of solution in a 96-well plate (Microplate, PS, 96 well, F-bottom).

### Photographs of the suspension

500 µL of solution was prepared in a capped glass tube and photographed, in front of a black curtain, using a Canon EOS 5D mark II camera equipped with an EF objective.

### Photo-stimulation and irradiation conditions

For all experiments except for Supplementary Fig. 11, the UV and blue photostimulation was performed on 100 µL sample in 96-well plate placed on the stage of an optical microscope (Axio Observer Z1, Zeiss) and irradiated using the microscope light source with UV (395/25 nm) and blue (480/30 nm) excitation filters. Under those conditions, the intensity received by the sample was measured using a powermeter (PM100D, Thorlabs GmbH) to be 26 mW·cm^−2^ and 40 mW·cm^−2^ for UV and blue irradiation, respectively.

For Supplementary Fig. 11, the UV irradiation (365 nm) was done with a LED irradiation system (CoolLED, PrecisExcite) equipped with an optical fiber that was placed at approximately 1 cm from the solution surface. Under that conditions, the intensity received by the sample was measured to be 24 mW·cm^-2^.

### Response to metal ions

A mixture of ATP (1 mM) and AzoDiGua (1 mM) in Tris HCl (50 mM, pH = 7.4) was freshly prepared according to the procedure described hereabove. After 10 min, 4 µL (resp. 20 µL) from an aqueous freshly-prepared 50 mM stock solution of metal ions (Cu(NO_3_)_2_, CaCl_2_, MgSO_4_, NaCl and KCl) was added to 100 µL (resp. 500 µL) of an ATP/AzoDiGua mixture and pipetted back and forth several times to reach a ratio [M^N+^]/[ATP] = 2. The same procedure was applied adding 4 µL of water in order to make sure that the 4% dilution didn’t affect the ATP/AzoDiGua aggregates. Specifically for Cu(NO_3_)_2_ ion, after adding the metal ion to the ATP/AzoDiGua mixture, the total volume of the solution was directly poured into a 96 well plate. We then observed the solution on an optical microscope (Axio Observer Z1, Zeiss) or acquired its fluorescence emission spectra.

### EPR spectroscopy

Continuous Wave Electron Paramagnetic Resonance experiments were performed at 110 K with a Bruker ELEXSYS E500 spectrometer operating at 9.26 GHz and equipped with a Bruker SHQE resonator and a Dewar Insert. The solutions were inserted in a quartz tube and then frozen by liquid nitrogen in the Dewar insert. The spectra were recorded under non-saturating conditions: a microwave power of 10 mW, a modulation amplitude of 0.6 mT and a modulation frequency of 100 KHz.

### Enzyme response characterization

Alkaline phosphatase enzyme (1 U·µL^−1^, equivalent to 0.5 µg protein·µL^−1^) was used as delivered from Sigma Alrich. A fresh ATP/AzoDiGua solution was prepared with the same procedure described hereabove. After 10 min of self-assembly, 5 µL (resp. 25 µL) of the enzyme solution was added to 100 µL (resp. 500 µL) to the ATP/AzoDiGua solution for the microscopy observations and fluorescence characterization (resp. turbidity study). The mixture was homogeneized by pipetting the mixture up and down several times. The optical microscope observation as well as the fluorescence studies were done right away after adding the enzyme. For Supplementary Fig. 19, the enzyme was previously denatured by a treatment at 90 °C for 30 min using a thermoblock (Eppendorf, Thermomixer C).

### UV-vis spectrophotometer

The absorption spectra were recorded with an Eppendorf BioSpectrometer.

### Supplementary information

Supplementary Information

Description of Additional Supplementary Files

Supplementary Movie 1

Supplementary Movie 2

## Data Availability

Most data are available in the main text and the Supplementary Information. Source data of the graphics of the main figures are available in a citable public repository (10.5281/zenodo.11658916) and can be accessed directly at the following link https://zenodo.org/records/11658916. Additional data can be obtained upon request to the corresponding author.
