# Supplementary material for: ATP/azobenzene-guanidinium self-assembly into fluorescent and multi-stimuli-responsive supramolecular aggregates
Source: Commun Chem. 2024 Jun 25;7:142. doi: 10.1038/s42004-024-01226-y (PMC11199595; doi:10.1038/s42004-024-01226-y)
Supplement: Supplementary file 1 — Supplementary Information [file 42004_2024_1226_MOESM1_ESM.pdf]

Supporting information for:

ATP/azobenzene-guanidinium self-assembly into fluorescent and multi-stimuli responsive supramolecular aggregates

Olivier Abodja,[1] Nadia Touati,[2] Mathieu Morel,[1] Sergii Rudiuk,[1] Damien Baigl[1,*]

[1] PASTEUR, Department of Chemistry, École Normale Supérieure, PSL University, Sorbonne Université, CNRS, 75005 Paris, France

[2]Chimie ParisTech, Université PSL, CNRS, Institut de Recherche de Chimie-Paris, PCMTH, 75005 Paris, France

* correspondence to: damien.baigl@ens.psl.eu

Contents:

1) Materials

2) Supplementary Note 1

3) Supplementary Figures 1–20

4) Supplementary References

1) Materials

Chemicals and reagents

Adenosine 5'-triphosphate disodium salt hydrate (ATP), Trizma hydrochloride solution (Tris HCl), Alkaline phosphatase from calf intestine (ALP, Roche Cat No. 10 713 023 001) were purchased from Sigma Aldrich. Milli-Q water (Millipore, 18.2 MΩ·cm) was used for all experiments. Azobenzene-containing symmetric divalent guanidinium compound (AzoDiGua) was synthesized as described previously[1]. Calcium chloride dihydrate ($CaCl_2$), Magnesium sulfate ($MgSO_4$), Potassium chloride (KCl), Sodium chloride (NaCl), Copper(II) nitrate trihydrate ($Cu(NO_3)_2$), Adenosine-5'-diphopshate sodium salt and Adenosine-5'-monophosphate disodium salt were purchased from Sigma Aldrich and used as delivered.

2) Supplementary Note 1: Self-assembly in complex media

ATP-AzoDiGua assembly was finally conducted in various complex media. For instance, ATP (1 mM) and AzoDiGua (1 mM) were mixed in Tris HCl buffer (pH = 7.4) in the presence or absence of a complex cocktail of 207 oligonucleotides (10 nM each nucleotide) coding for sharp triangle DNA origamis[2]. In both cases, we observed the immediate formation of the aggregates characterized by the appearance of turbidity and similar fluorescence emission spectra (**Figure S20A**). We also mixed ATP (1 mM) and AzoDiGua (1 mM) in Tris HCl buffer (pH = 7.4) in the presence of various amounts of Fetal Bovine Serum (FBS), a complex mixture of various biomolecules, including growth factors, proteins and vitamins, up to 10 vol%, the typical concentration used for cell culture. For all tested FBS amounts, aggregates appeared right after mixing ATP and AzoDiGua with a fluorescence nearly unaffected by the presence of FBS (**Figure S20B**). These results demonstrate the feasibility of the assembly in complex media containing a large number of biomolecules, including DNA and proteins, as well as in conditions compatible with cell cultures.

3) Supplementary Figures

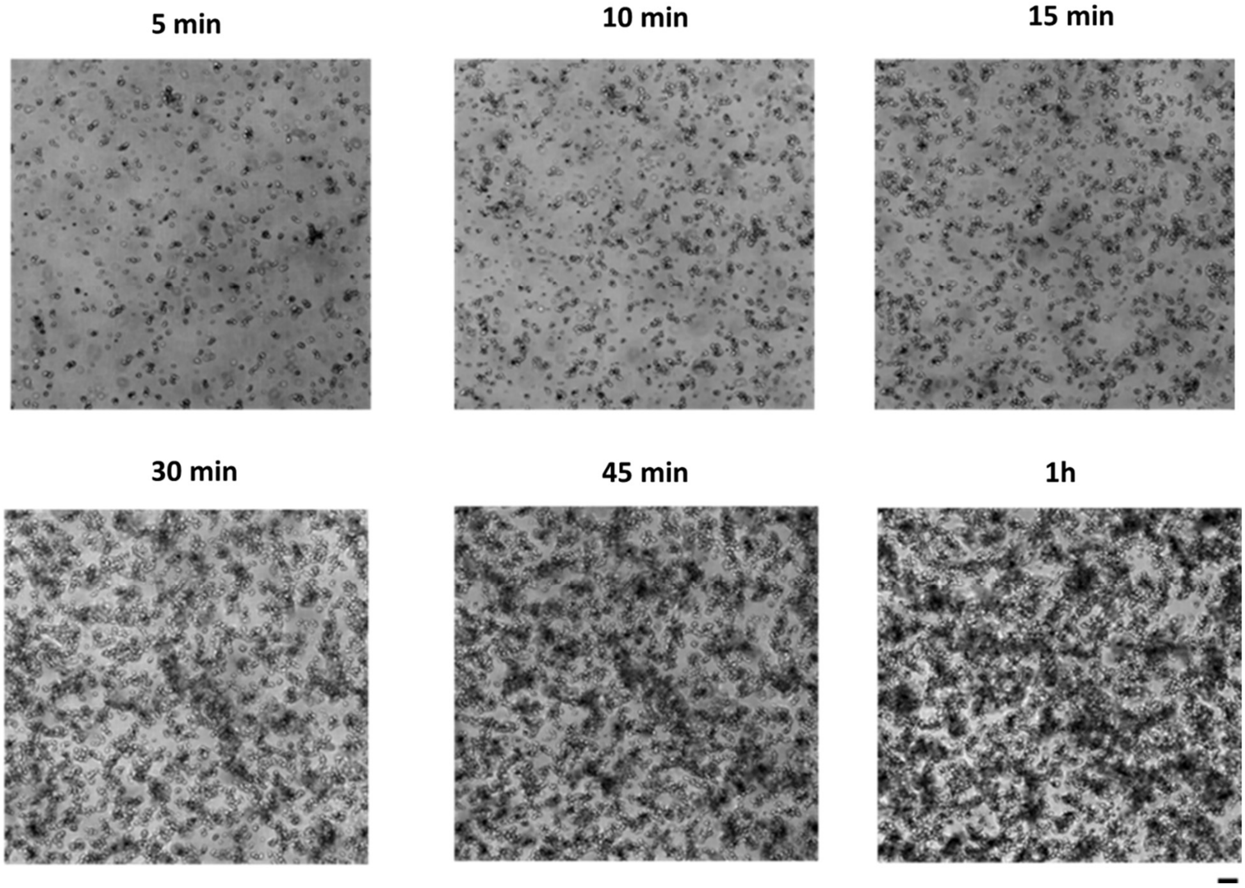

Supplementary Figure 1. Transmission optical microscopy image of ATP/AzoDiGua aggregates as a function of time after the assembly of ATP (1 mM) and AzoDiGua (1 mM) in 50 mM Tris HCl buffer (pH = 7.4) at room temperature. The scale bar is 20 μm.

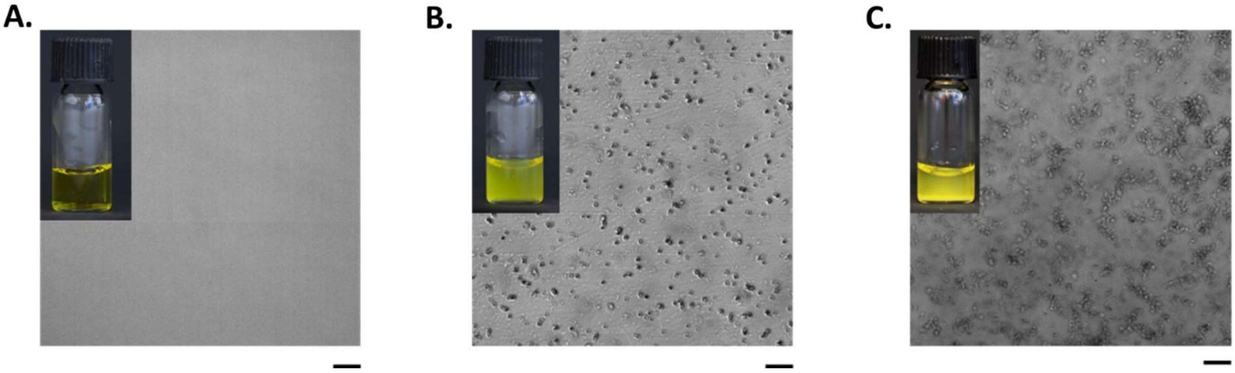

Supplementary Figure 2. Transmission optical microscopy images (main) and photographs (inset) of AzoDiGua (1 mM) after 10 min of self-assembly with 1 mM of adenosine monophosphate (AMP, A), diphosphate (ADP, B) or triphosphate (ATP, C). All solutions are in 50 mM Tris HCl buffer (pH = 7.4) at room temperature. All scale bars are 20 μm.

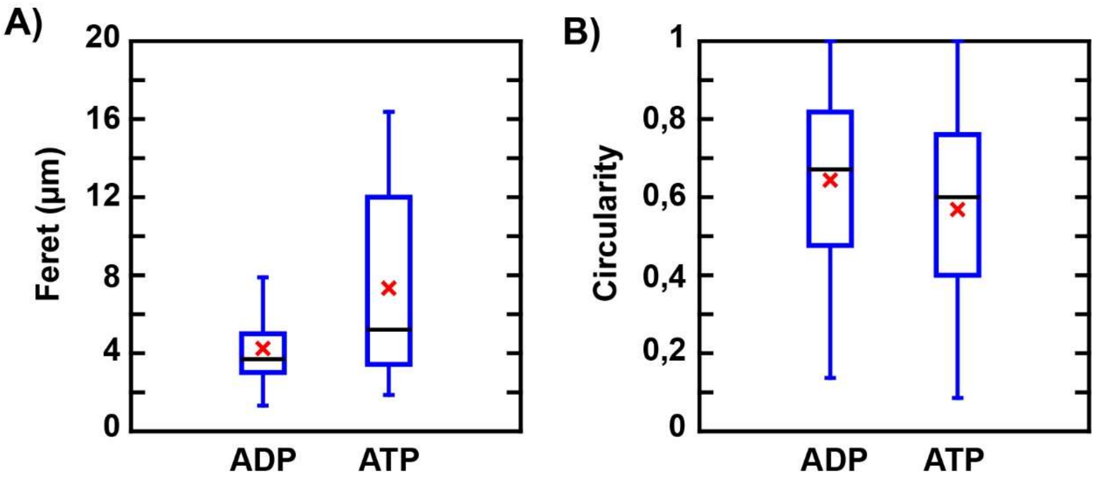

Supplementary Figure 3. Box plots of Feret (A) and Circularity (B) of the supramolecular aggregates obtained after 10 min of self-assembly of AzoDiGua (1 mM) with 1 mM of adenosine diphosphate (ADP) or triphosphate (ATP). The number of analyzed objects is *n* = 1044 (ADP) and *n* = 1078 (ADP). Blue box: range from first to third quartile, horizontal blue bars: minimum and maximum (outliers excluded); horizontal black line: median; red cross: mean. All solutions are done in 50 mM Tris HCl buffer (pH = 7.4) at room temperature.

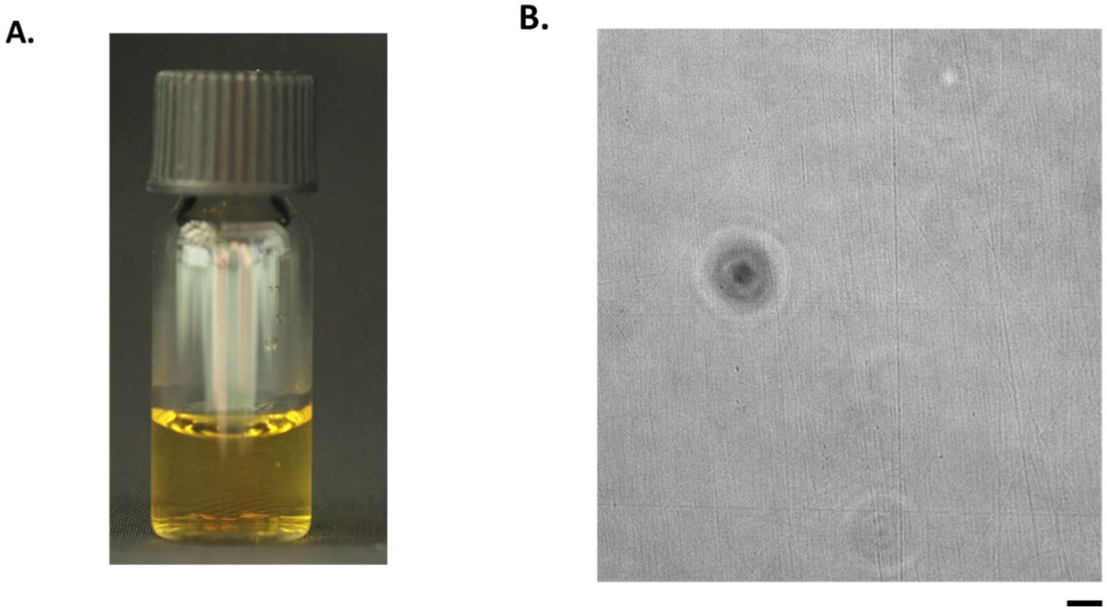

Supplementary Figure 4. Photograph **(A)** and transmission optical microscopy images **(B)** of a mixture of ATP (1 mM) and AzoDiGua (1 mM) after 10 min of incubation at room temperature in an acidic buffered solution (pH = 2.0). The scale bar is 20 µm.

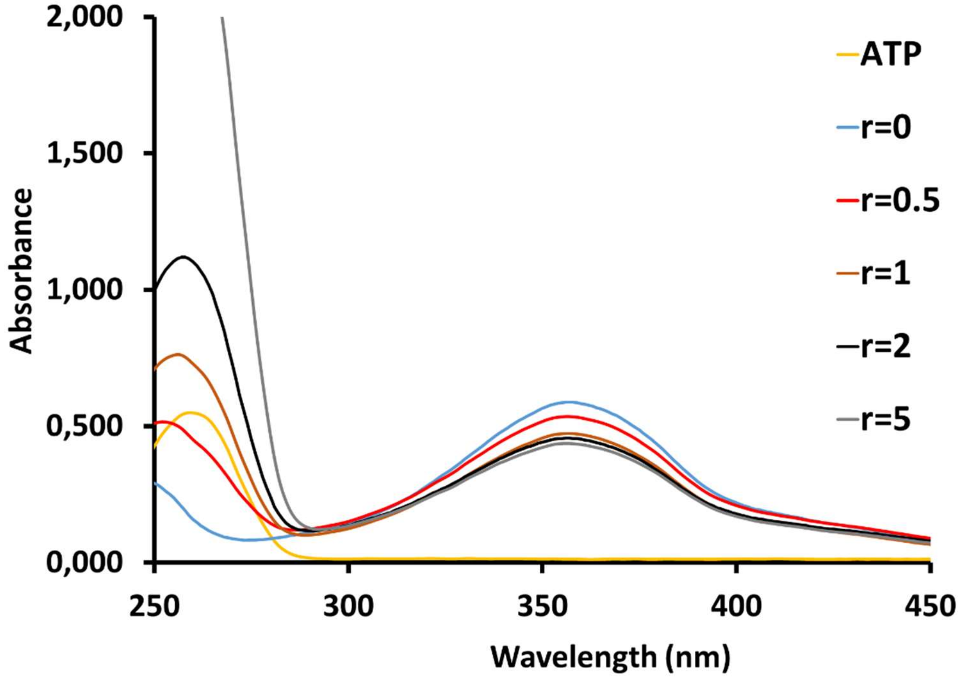

Supplementary Figure 5. Absorbance spectra of ATP alone (25 µM) or ATP/AzoDiGua with [AzoDiGua] = 25 µM and increasing ratios *r* = [ATP]/[AzoDiGua]. All solutions are done in 50 mM Tris HCl buffer (pH = 7.4) at room temperature.

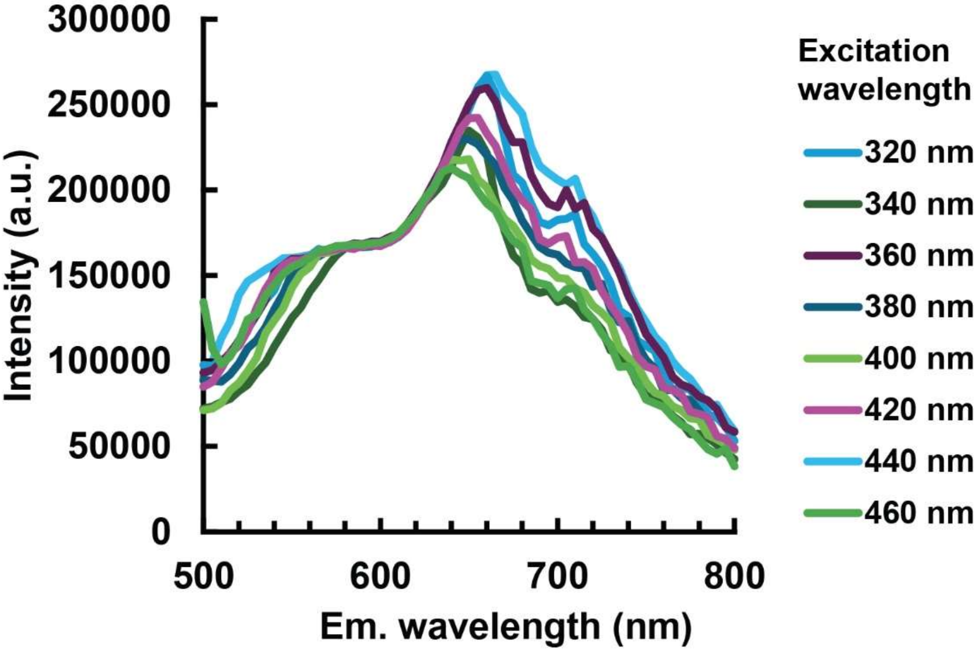

Supplementary Figure 6. Fluorescence emission spectra at varying excitation wavelengths of ATP/AzoDiGua aggregates ([ATP] = 1 mM, [AzoDiGua] = 1 mM, 10 min incubation). All solutions are done in 50 mM Tris HCl buffer (pH = 7.4) at room temperature.

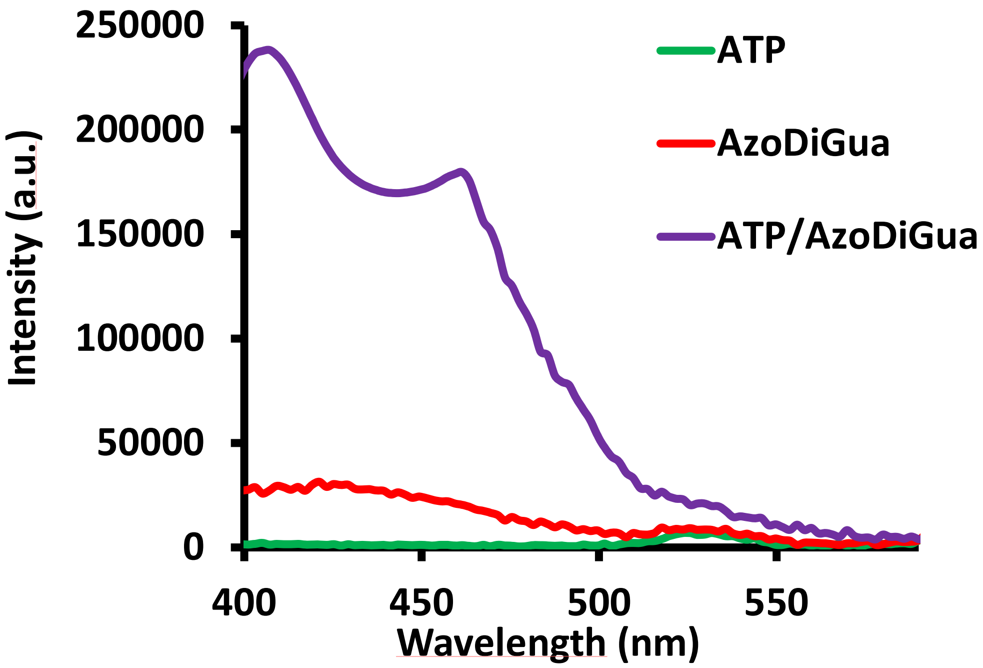

Supplementary Figure 7. Excitation spectra (Emission: 650 nm) of ATP alone (1 mM), AzoDiGua alone (1 mM) and ATP/AzoDiGua aggregates ([ATP] = 1 mM, [AzoDiGua] = 1 mM, 10 min incubation). All solutions are done in 50 mM Tris HCl buffer (pH = 7.4) at room temperature.

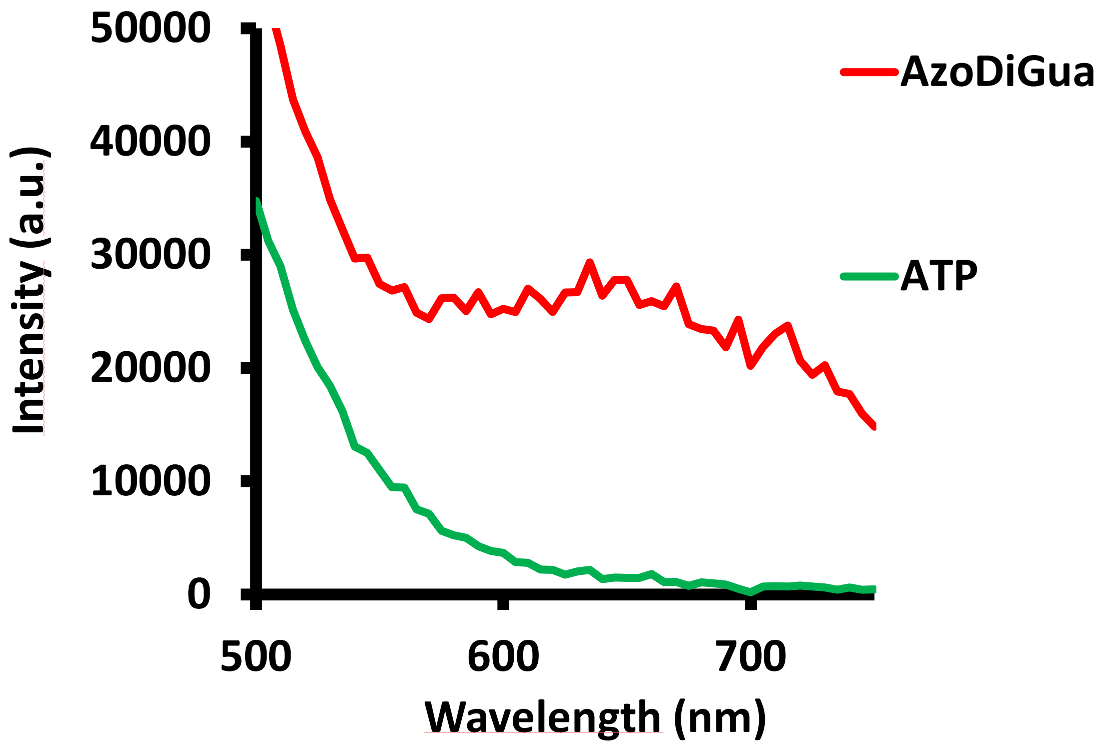

Supplementary Figure 8. Fluorescence emission spectra (Excitation : 400 nm) of AzoDiGua alone (1 mM) and ATP alone (1 mM). All solutions are in 50 mM Tris HCl buffer (pH = 7.4) at room temperature.

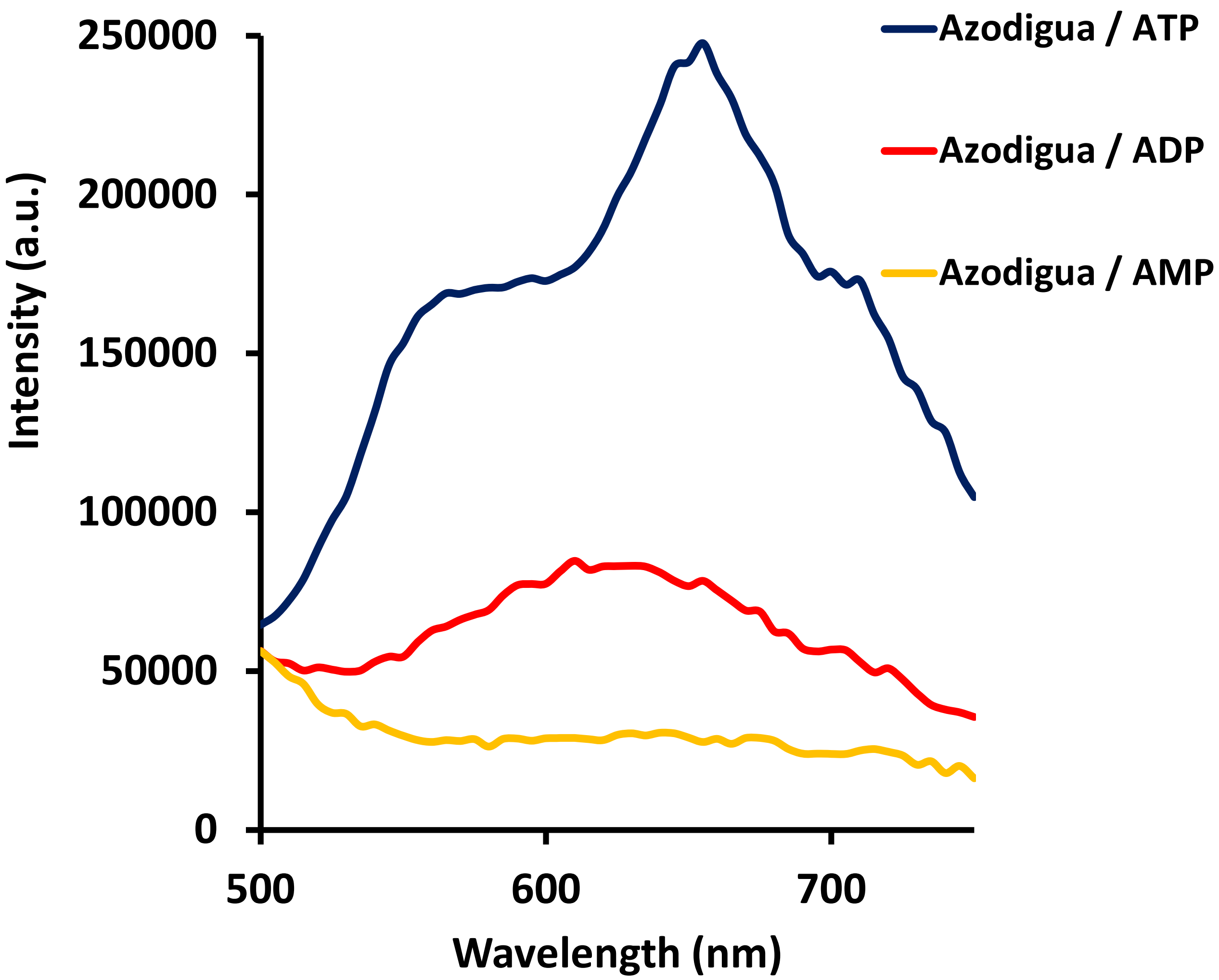

Supplementary Figure 9. Fluorescence emission spectra (Excitation: 400 nm) of AzoDiGua (1 mM) in the presence of ATP (1 mM), ADP (1 mM) or AMP (1 mM). All solutions are prepared in 50 mM Tris HCl buffer (pH = 7.4) at room temperature with 10 min incubation before characterization.

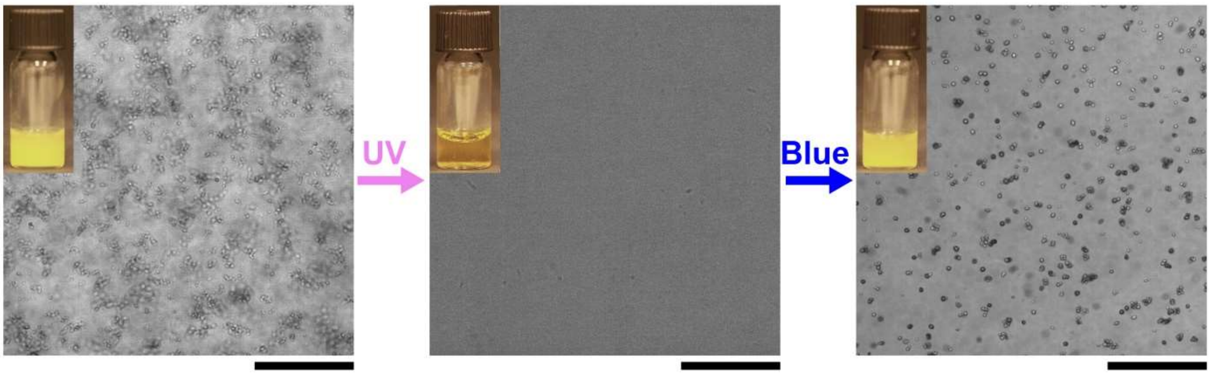

Supplementary Figure 10. Transmission optical microscopy images (main) and photographs (inset) of ATP/AzoDiGua suspension before (left) and after successive exposure to UV irradiation (395/25 nm, 26 mW·cm^{-2}) for 3 min (middle) and blue irradiation (480/30 nm, 40 mW·cm^{-2}) for 3 min (right). The initial suspension results from the self-assembly of ATP (1 mM) and AzoDiGua (1 mM) for 10 min. The scale bars are 50 μm. All solutions are prepared in 50 mM Tris HCl buffer (pH = 7.4) at room temperature.

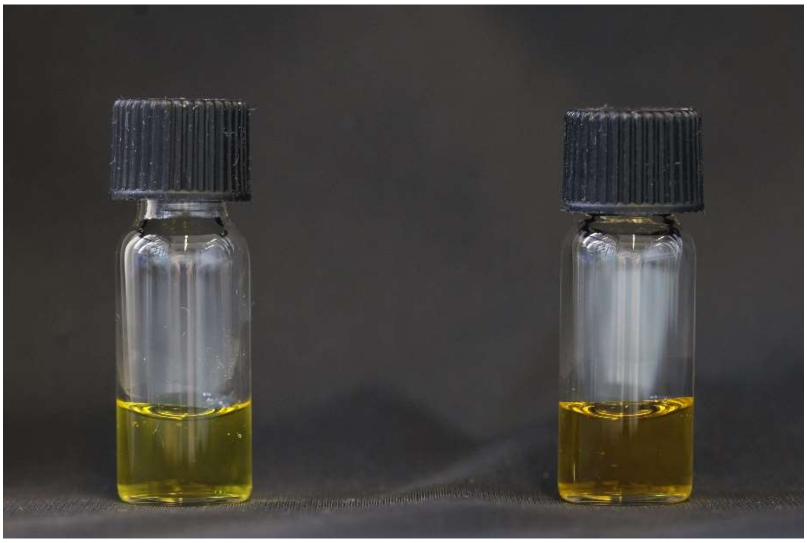

Supplementary Figure 11. Photograph of an AzoDiGua solution (1 mM) before (left) and after (right) UV irradiation (Excitation: 365 nm; 24 $mW.cm^{-2}$). The solutions are done in 50 mM Tris HCl buffer (pH = 7.4) at room temperature.

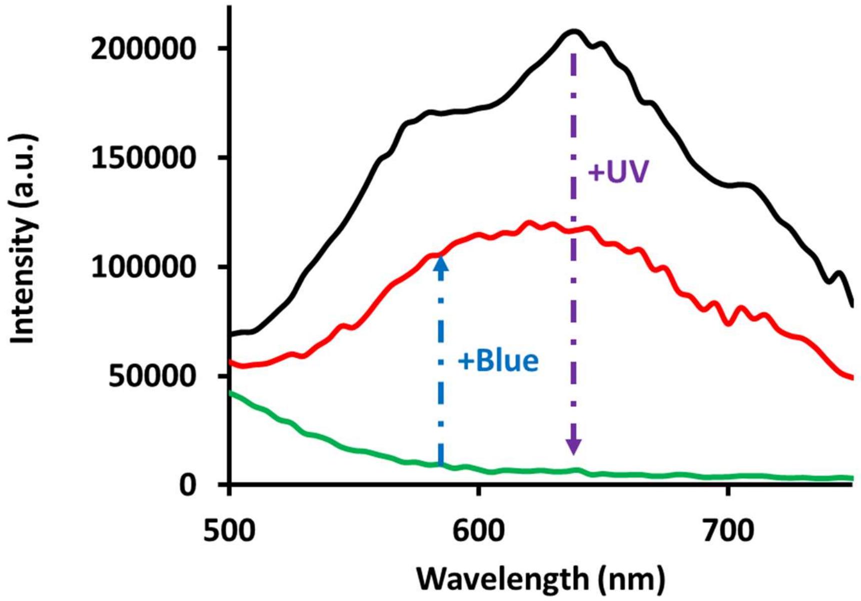

Supplementary Figure 12. Fluorescence emission spectra (Excitation : 400 nm) of ATP/AzoDiGua mixture ([ATP] = 1 mM, [AzoDiGua] = 1 mM) before (blue) and after successive irradiations by UV (395/25 nm; 26 $mW.cm^{-2}$, green) for 3 min and blue (480/30 nm; 40 $mW.cm^{-2}$, red) for 3 min. The solution was prepared in the regular conditions ([ATP] = 1 mM, [AzoDiGua] = 1 mM, 10 min incubation in 50 mM Tris HCl buffer (pH = 7.4) at room temperature).

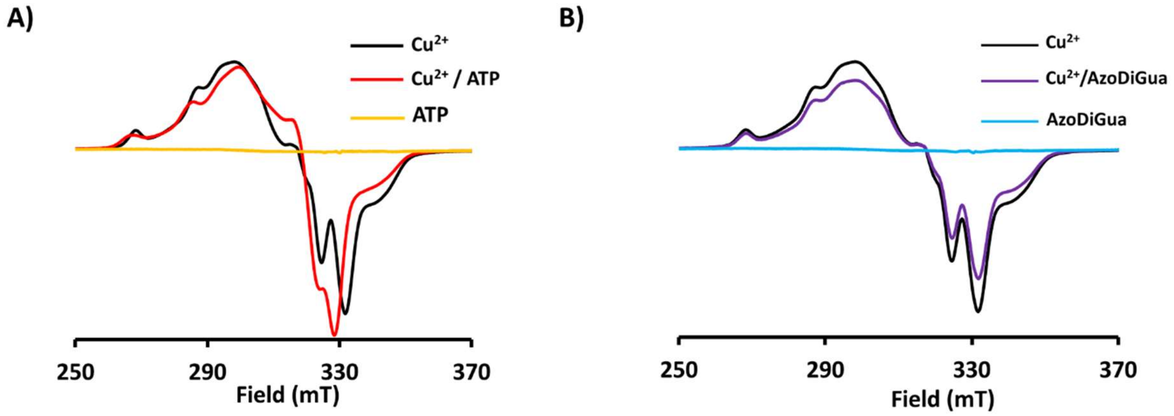

Supplementary Figure 13. A) EPR spectra (T = 110 K) of $Cu(NO_3)_2$ alone, ATP alone, and a mixture of ATP and $Cu(NO_3)_2$. B) EPR spectrum (T = 110 K) of $Cu(NO_3)_2$ alone, AzoDiGua alone, and a mixture of AzoDiGua and $Cu(NO_3)_2$. Conditions: [$Cu(NO_3)_2$] = 2 mM; [ATP] = 1 mM; [AzoDiGua] = 1 mM. All solutions are prepared in 50 mM Tris HCl buffer (pH = 7.4) at room temperature.

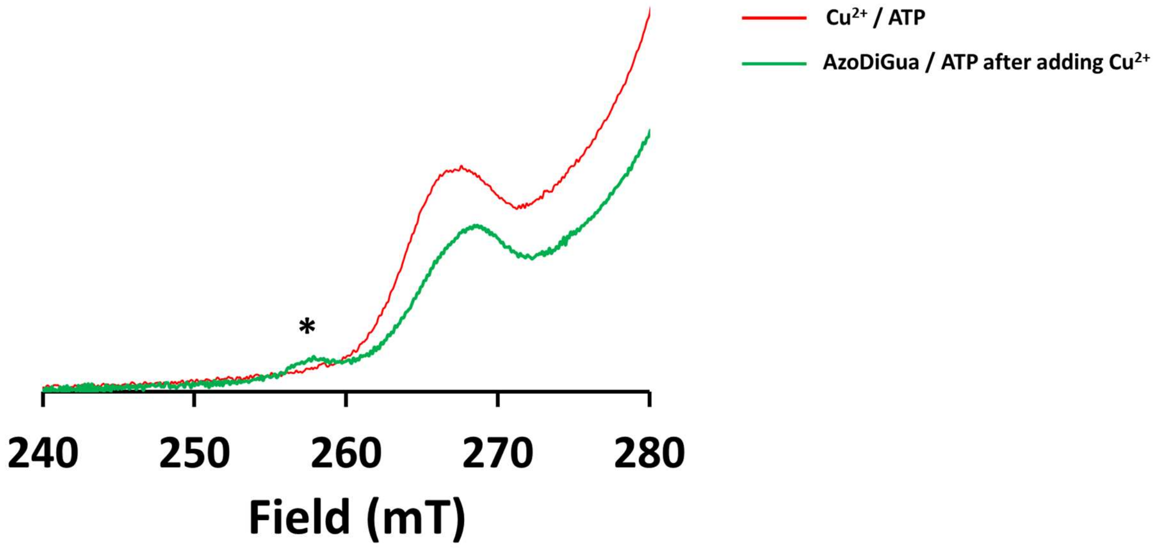

Supplementary Figure 14. EPR spectrum (T = 110 K) of a mixture of ATP and $Cu(NO_3)_2$ and ATP/AzoDiGua aggregates after dissolution by adding $Cu(NO_3)_2$. Conditions: $[Cu(NO_3)_2]/[ATP] = 2$; [ATP] = 1 mM; [AzoDiGua] = 1 mM. All solutions are prepared in 50 mM Tris HCl buffer (pH = 7.4) at room temperature. The asterisk indicates the appearance of a new peak.

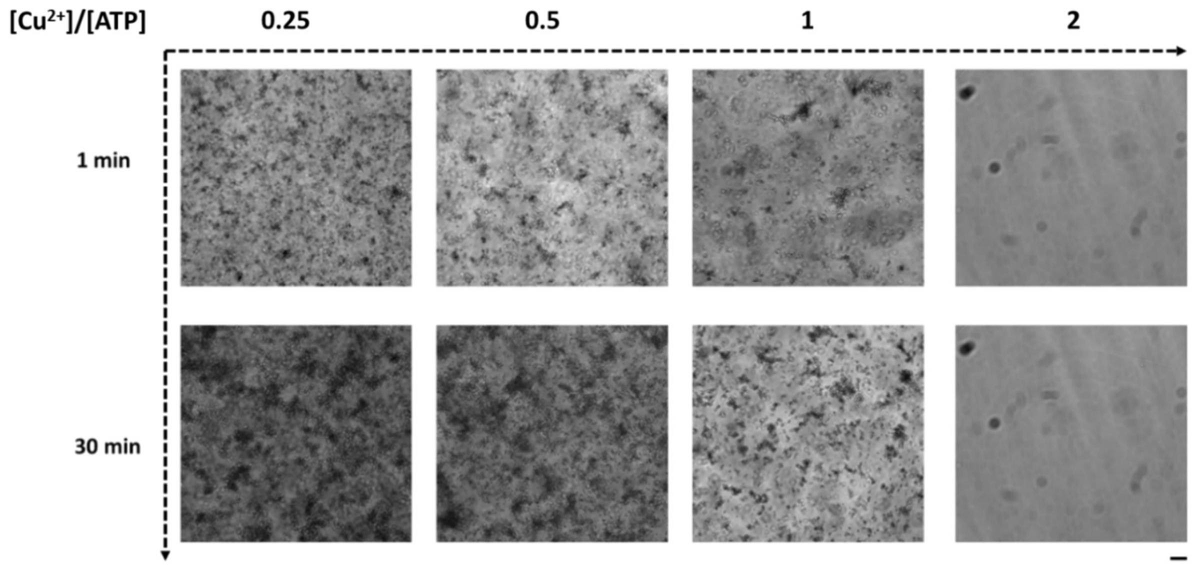

Supplementary Figure 15. Transmission optical microscopy images of ATP/AzoDiGua aggregates 1 min (top) and 30 min (bottom) after adding increasing ratios [Cu^{2+}]/[ATP]. The initial ATP/AzoDiGua suspensions were prepared in the regular conditions ([ATP] = 1 mM; [AzoDiGua] = 1 mM; 10 min incubation). The remaining pattern visible at [Cu^{2+}]/[ATP] = 2 corresponds to defects in the imaging set-up and not to actual aggregate materials. All solutions are prepared in 50 mM Tris HCl buffer (pH = 7.4) at room temperature. The scale bar is 20 μm.

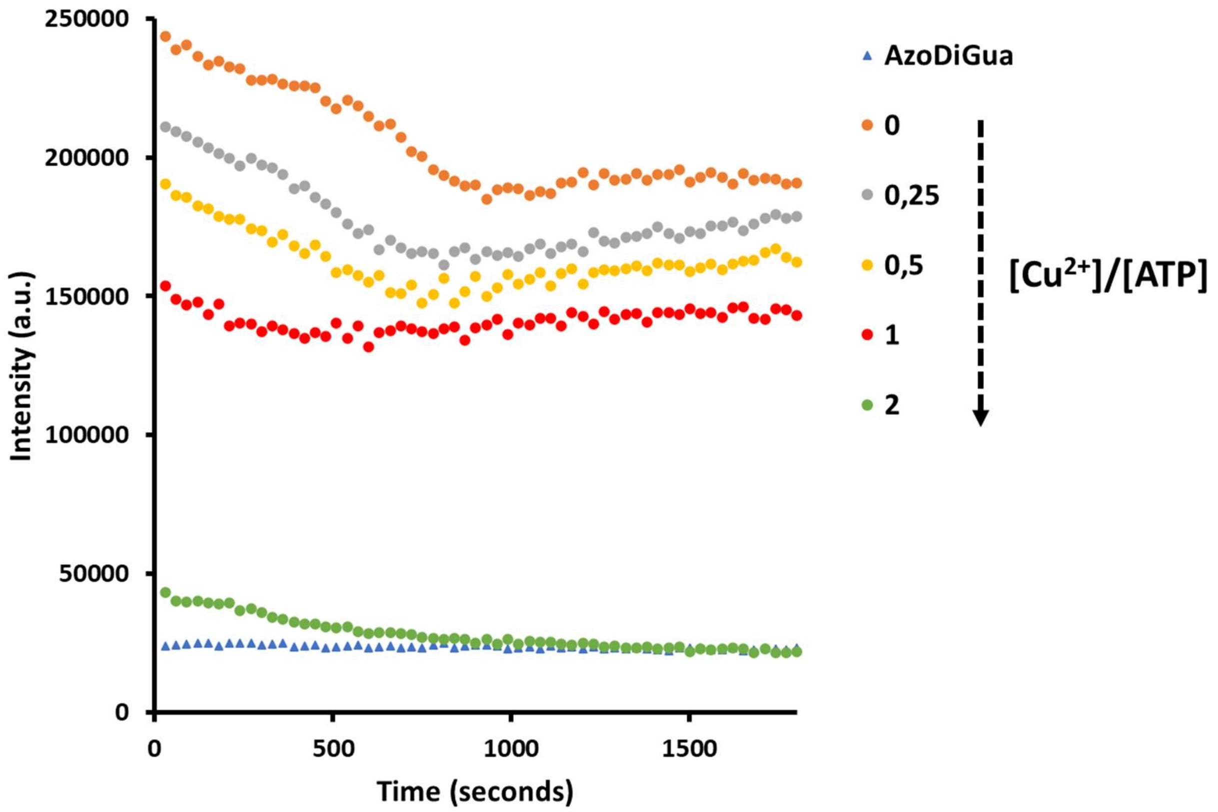

Supplementary Figure 16. Fluorescence emission at 650 nm (excitation 400 nm) over time of AzoDiGua alone (1 mM, triangles) and ATP/AzoDiGua aggregates (circles) for various amounts of added $Cu(NO_3)_2$ displayed as increasing ratios of $[Cu^{2+}]/[ATP]$. The ATP/AzoDiGua aggregates were prepared in the regular conditions ([ATP] = 1 mM; [AzoDiGua] = 1 mM). After 10 min of assembly, $Cu(NO_3)_2$ is added if needed and time is set to $t = 0$. Solutions are prepared in 50 mM Tris HCl buffer (pH = 7.4) at room temperature.

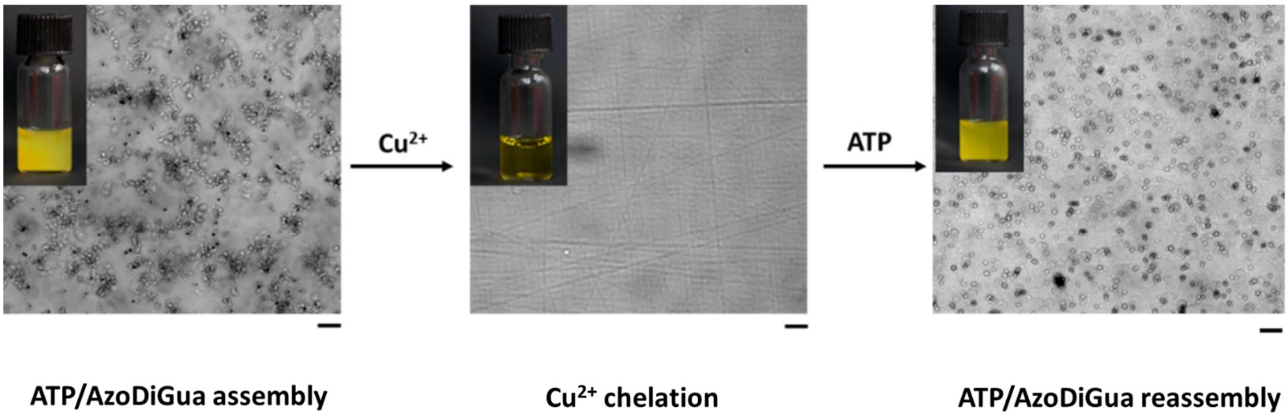

Supplementary Figure 17. Transmission optical microscopy images (main) and photographs (inset) of AzoDiGua/ATP aggregates before (left) and successive addition of $Cu(NO_3)_2$ ($[Cu^{2+}]/[ATP] = 2$) for 1 min (middle) and ATP ($[ATP]/[Cu^{2+}] = 3/2$) for 1 min (right). The initial ATP/AzoDiGua aggregates were prepared in the regular conditions (([ATP] = 1 mM; [AzoDiGua] = 1 mM; 10 min incubation). All solutions are prepared in 50 mM Tris HCl buffer (pH = 7.4) at room temperature. All scale bars are 20 μm.

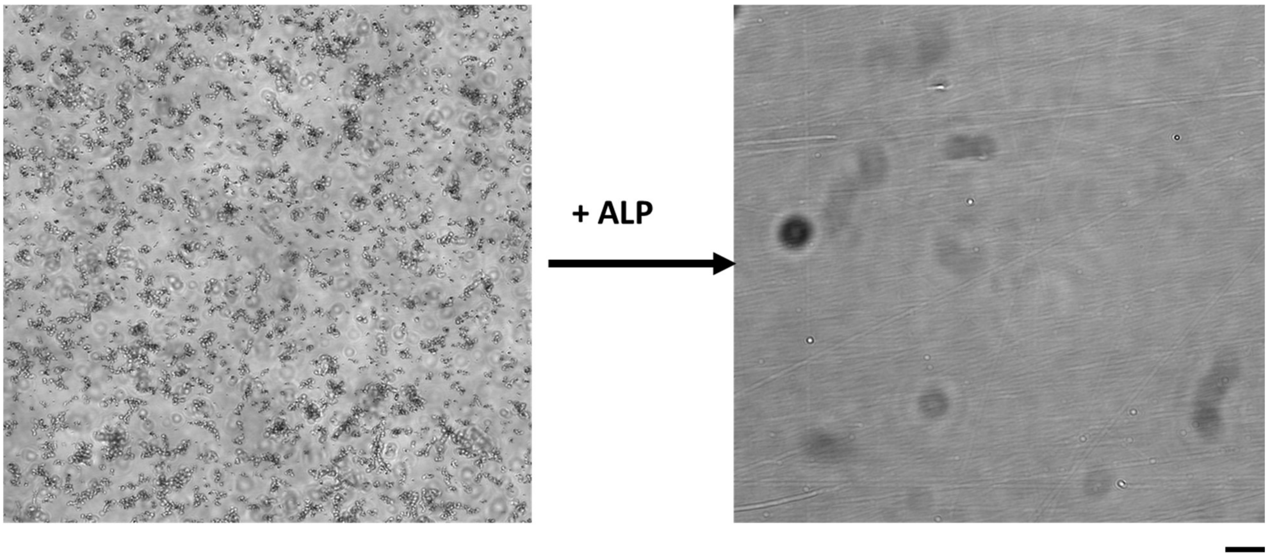

Supplementary Figure 18. Transmission optical microscopy images of ATP/AzoDiGua aggregates before (left) and after (right) adding ALP enzyme (50 U). The initial ATP/AzoDiGua aggregates were prepared in the regular conditions ([ATP] = 1 mM; [AzoDiGua] = 1 mM; 10 min incubation). The remaining pattern visible in the right image corresponds to defects in the imaging set-up and not to actual aggregate materials. All solutions are prepared in 50 mM Tris HCl buffer (pH = 7.4) at room temperature. The scale bar is 20 μm.

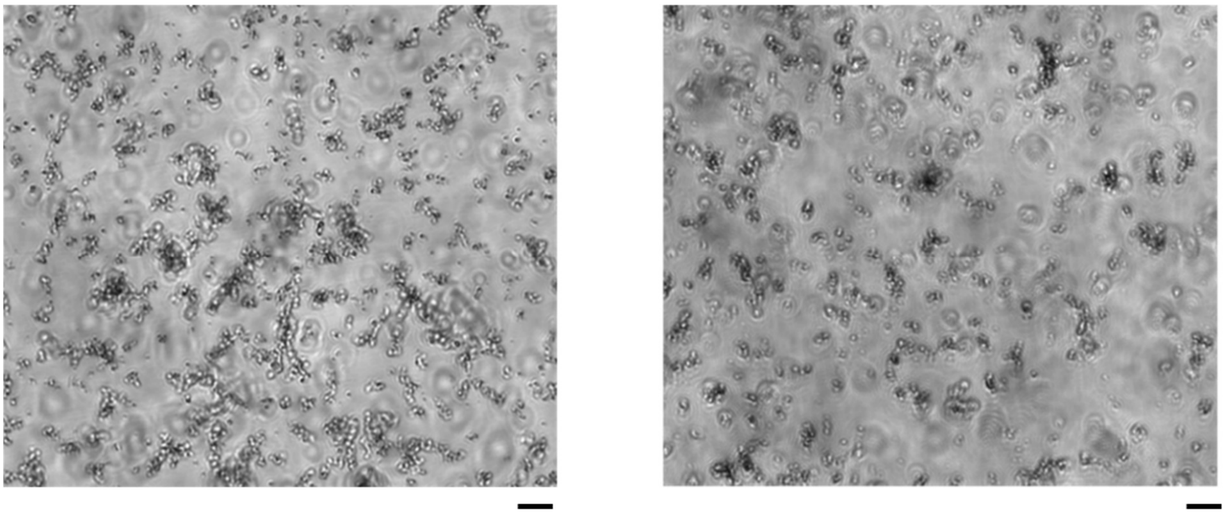

Supplementary Figure 19. Transmission optical microscopy images of ATP/AzoDiGua aggregates before (left) and after (right) adding ALP enzyme (50 U) previously heated at 90°C. The ATP/AzoDiGua aggregates were prepared in the regular conditions ([ATP] = 1 mM: [AzoDiGua] = 1 mM; 10 min incubation). All solutions are prepared in 50 mM Tris HCl buffer (pH = 7.4) at room temperature. The scale bar is 20 μm.

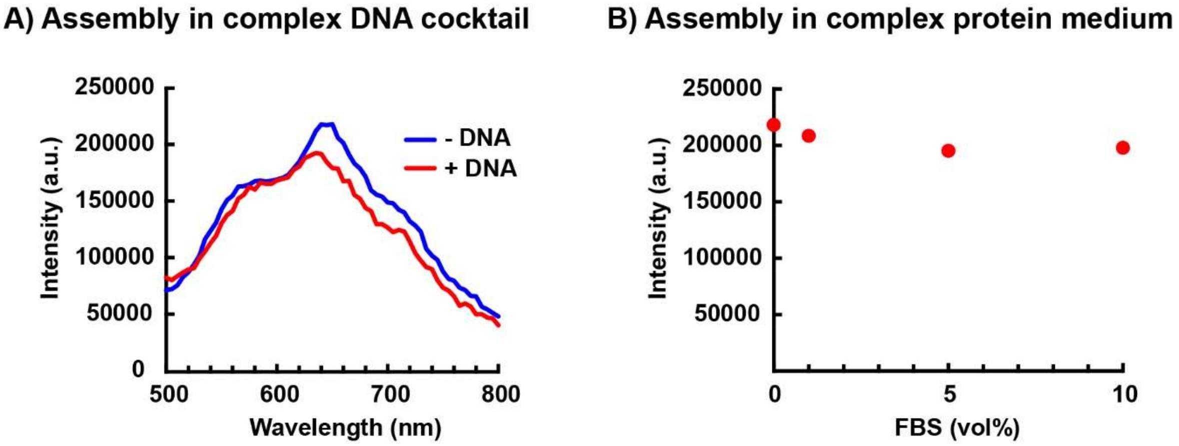

Supplementary Figure 20. A) Fluorescence emission spectra (Exc. 400 nm) after mixing ATP (1 mM) and AzoDiGua (1 mM) in the absence (-DNA) or presence (+DNA) a complex cocktail of 207 oligonucleotides (10 nM each nucleotide) coding for sharp triangle DNA origamis.[2] B) Fluorescence (Exc. 400 nm; Em. 650 nm) after mixing ATP (1 mM) and AzoDiGua (1 mM) with various volume fractions of Fetal Bovin Serum (FBS). All solutions are done in 50 mM Tris HCl buffer (pH = 7.4) at room temperature.

4) Supplementary References

1. Bergen, A. *et al.* Photodependent Melting of Unmodified DNA Using a Photosensitive Intercalator: A New and Generic Tool for Photoreversible Assembly of DNA Nanostructures at Constant Temperature. *Nano Lett* **16**, 773–780 (2016).

2. Rothemund, P. W. K. Folding DNA to create nanoscale shapes and patterns. *Nature* **440**, 297–302 (2006).
