# Supplementary material for: ATP/azobenzene-guanidinium self-assembly into fluorescent and multi-stimuli-responsive supramolecular aggregates
Source: Commun Chem. 2024 Jun 25;7:142. doi: 10.1038/s42004-024-01226-y (PMC11199595; doi:10.1038/s42004-024-01226-y)
Supplement: Supplementary file 2 — Description of Additional Supplementary Files [file 42004_2024_1226_MOESM2_ESM.pdf]

Description of Additional Supplementary Files

File name: Supplementary Movie 1

Description: Observation over time on an optical microscope (transmission mode) of ATP/AzoDiGua supramolecular aggregation at room temperature 30 seconds after mixing AzoDiGua (1 mM) and ATP (1 mM) in Tris HCl buffer (50 mM, pH = 7.4).

File name: Supplementary Movie 2

Description: Observation over time on an optical microscope (transmission mode) of ATP/AzoDiGua aggregates after successive UV (395/25 nm, 26 mW·cm-2) and blue (480/30 nm, 40 mW·cm-2) irradiations 10 minutes after mixing ATP (1 mM) and AzoDiGua (1 mM) in Tris HCl (50 mM) at room temperature.
